# Investigation on the contaminate of hand washing activities on the surface of environmental objects in intensive care unit

**DOI:** 10.1038/s41598-024-62529-7

**Published:** 2024-07-04

**Authors:** Fang-ying Tian, Xue-yu Wang, Hao-peng Meng, Jian-bang Kang, Ming Zhao, Hong-wei Wang

**Affiliations:** 1 Department of Nosocomial Infection Management, The Second Hospital of Shanxi Medical University, Taiyuan, 030001 Shanxi China; 2 School of Geology and Environment, Xi’an University of Science and Technology, Xi’an, 710054 Shaanxi China; 3 Department of Pharmacy, The Second Hospital of Shanxi Medical University, Taiyuan, 030001 Shanxi China; 4 Nursing College of Shanxi Medical University, Taiyuan, 030001 Shanxi China

**Keywords:** Intensive care units, Taps, Droplets, Contaminate situation, Microbiology, Medical research

## Abstract

To detect the contaminate of faucets in hospitals and the splash during hand washing, and to explore the reasonable layout of hand washing pools. Two faucets with roughly the same spatial layout in the ICU of a third-class first-class general hospital were selected, and the farthest splashing distance and specific splashing points were measured by color paper. Samples were detected by ATP detection technology and routine microbial detection method, and the contaminate of faucets was analyzed. After 72 h of daily hand-washing activities, the furthest distance to the splash point was about 100 cm around the faucet, and the place 40–110 cm around the faucet was contaminated seriously. The farthest distance that the splash point reached was about 80 cm around the faucet with the center of the circle, and the area 40–60 cm around the faucet was heavily contaminated. The distance from the water outlet of the long handle and the short handle faucet to the detection point had a high negative correlation (*r* = − 0.811, *P* < 0.001) and a moderate negative correlation (*r* =  − 0.475, *P* = 0.001) with the number of splash points, respectively. The qualified rates of ATP detection and microbial culture were 25% and 15%, respectively. Pseudomonas aeruginosa, Staphylococcus epidermidis, and other pathogenic bacteria were detected in the water outlet of the faucet and the surrounding environment. Safe hand hygiene facilities are one of the important guarantees of hand hygiene effect. Clean objects and objects related to patients should not be placed within 1 m range near the water outlet of faucet. Anti-splash baffle should be installed as much as possible when conditions permit to reduce the contaminate caused by splash during hand washing.

## Introduction

Water-borne infection disease is the general name of all diseases related to water use^1^, In the United States, about 25% of hospital-related infections are caused by water-borne pathogens^2^. By summarizing 52 studies, it is found that the increasing antimicrobial rate has focused on the sink drainage system, which is the repository of hospital-acquired bacteria colonization and infection^3^ The contamination of hospital sinks by microbial pathogens poses a serious potential threat to patients, and our understanding of sink colonization dynamics is largely based on infection outbreaks^4^. Domestic scholars have reviewed the relevant literature on tap water in public places and found that hospital tap water is the most contaminated^5,6^. There are many ways of water-borne infection in hospitals, such as inhaling aerosol containing pathogens, choking contaminated water, and direct or indirect contact with contaminated water to cause infection^7^. Kotay’s research found that the number of hospital infection outbreaks caused by hand washing increased dramatically, so it was generally believed that hand washing pool was the main host of antibiotic-resistant pathogens in patient care area^8^. Hajar’s research proves that hand washing causes gram-negative bacilli to spread frequently from the drainage pipe of the hospital’s colonization sink to cover clothes and hands^9^. Yui's research found that more than 1000 droplets were formed during the 30 s washing process, and the diffusion distance of droplets was found to be more than 2 m. However, the number of droplets formed in the process of washing hands is significantly reduced and the diffusion distance is shortened through the improved washbasin^10^. Washing hands with running water is a key barrier to avoid cross infection. The knowledge of the hazards related to the location of the pool has been neglected for more than 45 years, until the outbreak of neonatal pseudomonas disease in Belfast in 2012 forced people to make changes. It is not only to detect whether there are pathogens in the water at the outlet of the sink, but also to actively seek comprehensive methods to minimize risks^11^. The surface of contaminated environmental organisms and the hands of health professionals in health care institutions are important carriers for the spread of pathogens, and are also important incentives and hidden dangers for the spread of multi-drug resistant bacteria and the outbreak of hospital infection. Faucet is a bridge connecting people with water. Contaminated faucets will directly pollute the water, and then pollute the hands of medical staff. Medical staff will be prone to cross-infection when they conduct medical activities for patients. Hota and other research experiences show that in addition to ensuring a sufficient number of hand-washing basins, the placement and design of hand-washing basins is a crucial factor in the design of hospital wards, which is particularly important in intensive care units such as intensive care units^12^. Liao Dan and other studies show that faucets are the main source of indoor contaminate, so attention should be paid to the cleanliness of faucets and their surrounding environment, and cleaning items and items related to patients should not be placed within 1 m^13^. The results of a systematic review show that sinks and faucets are the common sources of nosocomial infection outbreaks of Pseudomonas aeruginosa around the world^14^ Considering the possibility of splashing and spreading of contaminated droplets, it is reasonable that people or patients may be directly contaminated when washing their hands. Through reasonable experimental design, this study analyzes the possibility of droplet diffusion and contaminate to the surrounding environment in the daily hand washing process of ICU medical staff in a tertiary general hospital, and compares the splashing distance of droplets when washing hands with long handle and short handle faucets commonly used in hospitals, and the contaminate of the surrounding environment within the scope of faucet and droplet diffusion, so as to provide a basis for discussing the rational layout of hand washing pools and formulating infection prevention and control measures.

## Methods

### Materials and methods

#### Test site

Two sinks with similar spatial structure and different distances between faucets and sinks are selected in ICU of a tertiary general hospital. Sinks A and B are located in different rooms of surgical ICU. Sink A is located in three rooms, with a short handle and a water outlet 22 cm; away from the sink. Washball B is located in a quadruple room with a long handle and a water outlet 33 cm away from the sink. Because the quadruple room is L-shaped, the spatial positions of the two washbasins are roughly similar. The location of hand-washing pool A treats surgical patients, while the location of hand-washing pool B treats only neurosurgical patients. By inquiring the statistics of the hospital in previous years, their bed utilization rates were 98.43% and 99.07% respectively. During the experiment, there was no empty bed in both rooms, and a nurse was routinely deployed. Due to the particularity of sharing doctors in ICU and ward, the influence of doctor-nurse ratio was not considered in this study.

#### Materials and reagents

Self-adhesive label (hereinafter referred to as chromogenic paper) that turns red when it meets water, hand-held ATP bioluminescence detector of System SURE Plus, and related reagent detection stick containing luciferase, physiological saline cotton swab, nutrient agar and blood plate.

#### Experimental method

The test is divided into three stages, each stage lasts 72 h, and the water pressure is adjusted in advance and controlled at 0.1Mpa.

**Figure 1:**
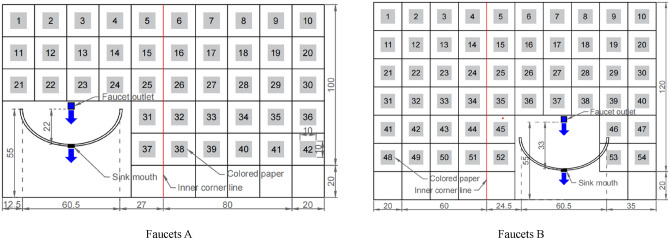
Schematic diagram of color paper pasting and sampling at faucets (**A**) and (**B**).

- (1) Testers put colored paper in the vertical and horizontal directions of the faucet to test the farthest splashing range of the faucet;
- (2) according to the farthest splash range, delimit the test range of the faucet vertically and horizontally by 20 cm  ×  20 cm, post 10 cm  ×  10 cm color paper in each test area, observe and record the times of hand washing on the spot, and eliminate the abnormal splash points (splash points not caused by hand hygiene) in statistical analysis (even pieces cannot be counted); See Fig. 1 for the schematic diagram of color paper pasting and sampling.
- (3) Calculate the distance from the faucet outlet to the center of the color paper. The research results show that the nearest distance from the faucet outlet of sink A is 24.69 cm and the farthest distance is 106.44 cm, while the nearest distance from the faucet outlet of sink B is 36.23 cm and the farthest distance is 101.85 cm. The distances are divided into four ranges: 20–40 cm, 40–60 cm, 60–80 cm and 80–110 cm.

#### Method of sampling

ATP fluorescence detection: use the System SURE Plus hand-held ATP fluorescence detector and its swab to smear on the surface of the object vertically and repeatedly, and continuously collect the area of four 5 cm × 5 cm sterile plates with a total area of 100 cm^2^. Insert the detection tube into the hand-held ATP biological fluorescence detector and read the value within 15 s. The result is expressed as relative luminous unit value (RLU). Cotton swab smear method: the sampling area and method are the same as ATP fluorescence detection, and the sampled cotton swab head is cut into a test tube containing 10 ml physiological saline in an aseptic way and sent to the microbiology laboratory for inspection.

#### Cultivation and detection methods

ATP fluorescence detection: after sampling, put the sampling stick in the detection instrument and read the RLU value. Plate counting method of total bacterial count: fully shake the sampling solution with a vortex oscillator, take 0.5 ml and inoculate it into a common nutrient agar plate (Φ9 cm) and culture it at (36 ± 1) °C for 48 h, then count the colonies. Total number of colonies (CFU/cm^2^) = number of colonies on the plate × dilution multiple/sampling area, and calculate the total number of colonies.

#### Result evaluation

The differences of three results, namely, the number of splash points of faucets A and B in the sink, the RLU value of ATP fluorescence detection method and the total number of colonies, were compared. The total number of bacterial colonies on the surface of the object was ≤ 5 cfu/cm^2^, and the RLU value of ATP fluorescence detection was < 30. The number of splash points was centered on the outlets of faucets A and B, and the Euclidean distance from the center of the center to the center of the color paper was calculated($\rho =\sqrt{{({x}_{2}-{x}_{1})}^{2}+{({y}_{2}-{y}_{1})}^{2}+{({z}_{2}-{z}_{1})}^{2}}$)), Analyze the linear correlation between the distance and the number of splash points or curve fitting, and analyze the correlation between the number of splash points and the RLU value and the total number of colonies of ATP fluorescence detection method respectively.

#### Statistical analysis

SPSS 26.0 was used for statistical analysis. The results showed that the splash points at the outlets of faucets A and B did not obey the normal distribution, and nonparametric test (Wilcoxon rank sum test of two independent samples) was adopted. Spearman rank correlation is used to analyze the correlation between distance and splash points. The counting data were expressed in *n* or %, and the Fisher’s exact probability method was used for the comparison between groups.

## Results

### Farthest splashing distance

After the colored paper was placed for 72 h, the tester found a splash point at the outlet of faucet A in the sink about 120 cm horizontally and 60 cm vertically. Splash points were found at the water outlet of faucet B of sink about 140 cm horizontally and 80 cm vertically.

### Splash points of sink A and B at different distance and their correlation analysis

**Figure 2:**
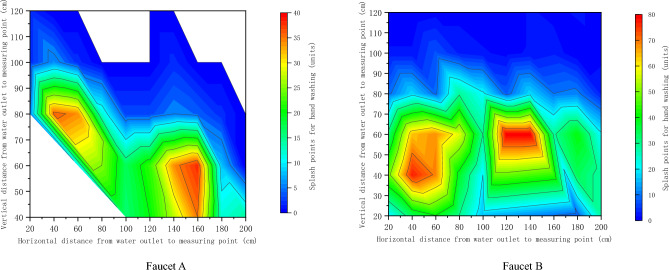
Contour map of washing and splashing points at faucets (**A**) and (**B**) (plane distance).

**Figure 3:**
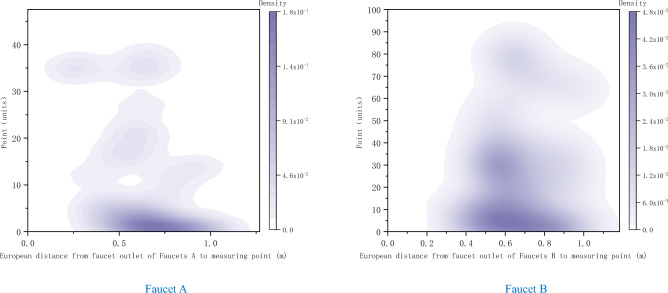
Nuclear density diagram of the number of splashing points at faucets (**A**) and (**B**) (spatial distance).

There were 361 splash points in sink A, and there were no abnormal splash points. A total of 1168 splash points were generated in sink B, and 2 abnormal splash points were excluded (caused by dripping towel); The distribution range of splash points of hand-washing basin A and B is shown in Fig. 2 and Fig. 3. The results of plane distance show that the horizontal distance of hand-washing basin A is 30–60 cm, 140–170 cm, and the vertical distance is 70–90 cm, while the horizontal distance of hand-washing basin B is 30–70 cm, 140–170 cm, and the vertical distance is 110–150 cm. The spatial distance results show that 40–70 cm away from the faucet outlet of washbasin A is the concentrated area of splash points, with 0–10 splash points, 70–100 cm away from the faucet outlet of washbasin B is the concentrated area of splash points, and 50–70 cm is the concentrated area with 20–50 splash points.

Splash points of sinks A and B at different distances do not obey the normal distribution. After Wilcoxon rank sum test, the P value is 0.001 < 0.05, which shows that there are significant differences between splash points of sinks A and B. Through Spearman rank correlation, the correlation analysis between the distance from the faucet outlet to the detection point and the number of splash points in the sink A and B was made. The results showed that the distance from the faucet outlet to the detection point in the sink A and B was negatively correlated with the number of splash points (r = − 0.475, P = 0.001) and negatively correlated with the number of splash points (r = − 0.811, P < 0.001) respectively. See Table 1 for details.

**Table 1:** Statistical analysis results.

Faucets		Euclidean distance(m)				Test of normality		Non-parametric test		
		[0.2,0.4]	(0.4,0.6]	(0.6,0.8]	(0.8,1.0]	*W*	*P*	Average value	*Z*	*P*
Faucets A	Splash points	84	113	150	14	0.729	< 0.001	38.119	3.239	0.001
Faucets B		80	779	298	11	0.833	< 0.001	56.574		
Non-parametric test of different distances	*Z*	–	3.849	1.949	1.835					
	*P*	–	< 0.001	0.051	0.006					

### ATP test results and plate reading results at different sampling points

**Figure 4:**
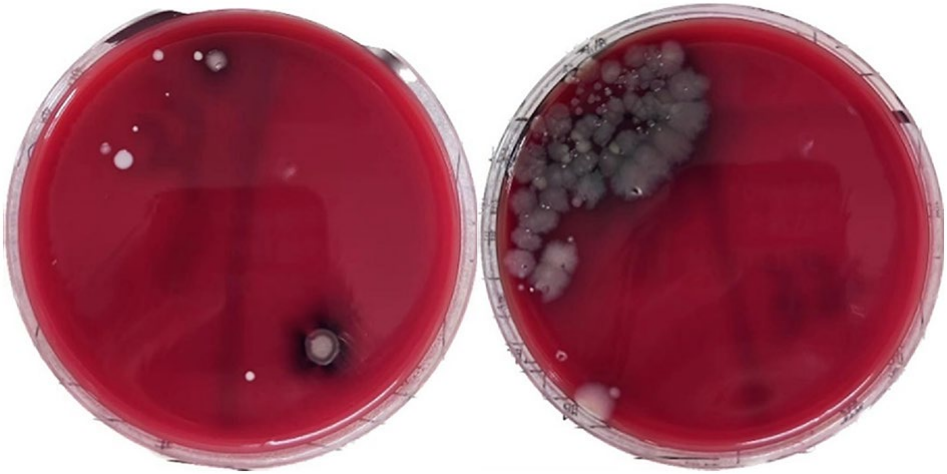
Results of partial blood plate coating.

Pseudomonas aeruginosa was detected in the water outlets of faucets A and B of the sink, and the number of colonies was 80 CFU/cm^2^ respectively, which could not be counted. Both of them did not meet the Class II environmental requirements in the Hygienic Standard for Hospital Disinfection (GB15982-2012): the total number of bacterial colonies on the surface of the object was ≤ 5 cfu/cm^2^; The qualified rate of ATP detection is 25%, and the qualified rate of routine microbial detection is 15%. See Table 2 for ATP detection results and plate reading results of each sampling point. The results of partial blood plate coating are shown in Fig. 4.

**Table 2:** ATP detection and microbial culture results at sampling points A and B of faucet.

Sample point	Detected bacteria	ATP fluorescence detection		Microbial detection	
		Relative light unit (RLU)	Qualified/unqualified	Colony number (CFU/cm^2^)	Qualified/unqualified
Faucets A					
A0	Pseudomonas aeruginosa Staphylococcus epidermidis	219	Unqualified	80	Unqualified
A1	Staphylococcus epidermidis Bacillus pumilus	34/45	Unqualified/Unqualified	10/15	Unqualified/Unqualified
A2	Staphylococcus epidermidis	75/87	Unqualified/Unqualified	20/28	Unqualified/Unqualified
A3	Staphylococcus epidermidis	23/39	Qualified/Unqualified	5/10	Qualified/Unqualified
A4	Staphylococcus epidermidis	12/30	Qualified/Qualified	3/7	Qualified/Unqualified
Faucets B					
B0	pseudomonas aeruginosa	791	Unqualified	Unable to count	Unqualified
B1	Staphylococcus epidermidis	3/27	Qualified/Qualified	0/53	Qualified/Unqualified
B2	Micrococcus gamboge Staphylococcus epidermidis	128/174	Unqualified/Unqualified	30/47	Unqualified/Unqualified
B3	Staphylococcus epidermidis	593/627	Unqualified/Unqualified	175/213	Unqualified/Unqualified
B4	Staphylococcus epidermidis	146/179	Unqualified/Unqualified	50/75	Unqualified/Unqualified

A0: faucet outlet of sink A, A1: 10 cm away from the outlet, A2: 30 cm away from the outlet, A3: 60 cm away from the outlet, A4: 100 cm away from the outlet; B0: faucet outlet of washbasin B, B1: 10 cm away from faucet, B2: 30 cm away from faucet, B3: 60 cm away from faucet, B4: 100 cm away from faucet and B5: 120 cm away from faucet.

## Discussion

In this study, we investigated and analyzed the droplet splash range of long-neck faucet and short-neck faucet when washing hands. It was found that the distance between faucet and outlet affected the droplet splash range, and the difference between them was statistically significant. When the distance between the water outlet and the detection point is equal, the number of splash points generated by hand-washing pool B is more than that of hand-washing pool A, and the farthest splash distance generated by hand-washing pool B is 100 cm, while that generated by hand-washing pool A is 80 cm. The results show that the long-neck faucet produces more splash points when washing hands than the short-neck faucet, and the splash covers a wider range. Wash your hands should avoid too many water droplets splashing, because the protective effect of hand hygiene is two-way. Medical staff should not only avoid being contaminated by patients, but also protect patients from being infected by contaminated instruments or contaminated medical staff’s hands. The splashing points are the most in the range of 40–80 cm centered on the faucet outlet. If cleaning articles and sterile articles are placed in this range, they will be easily contaminated, so it is forbidden to place cleaning articles and sterile articles in this range. According to the distribution of splash points, splash droplets scatter around with the faucet as the center. Therefore, if the washbasin is close to the bed unit, baffles or partitions should be installed as far as possible to prevent splashing. If there is no barrier between the sink and these surfaces, bacteria may transfer to the patient’s bed. The alarming increase in the number of hospital infection outbreaks related to hand-washing sinks has led to widespread recognition that sinks are the main repository of antibiotic-resistant pathogens in patient care areas. Hand washing is considered as one of the most important ways to reduce the spread of infectious microorganisms in the world. Previous studies have shown that the frequency of hand washing by health care workers is usually low. Unfortunately, despite the efforts of strengthening monitoring and education, such interventions often have little effect^15^. Patients in German Pediatric Oncology Ward had an outbreak after inhaling contaminated aerosol and touching patients with contaminated hands^16^. In a simulation experiment^17^ , a large number of droplets were produced within 30 min after the faucet was used. Because of the contaminate of the sink, these droplets contained pathogenic microorganisms, and after they spread to the surface of the object, these droplets-related microorganisms still survived, posing a threat to the surface of the object around the sink. It was found that GFP- E.coli dispersion was detected during the use of the faucet by using a settling plate or an impact and filtered air sampling method. It was not detected when the faucet was not used. This discovery also means that when the faucet outlet is facing the drain of the sink, the shearing force of the water flowing into the sink will lead to the diffusion of bacteria, while the scattered GFP- Escherichia coli was detected during the use of the faucet, but not at the subsequent time point (30 min later), which indicates that the scattered cells are related to larger and heavier droplets, and these droplets will quickly settle to the surface due to gravity, rather than the size of aerosol trapped in the air. In this study, Pseudomonas aeruginosa was detected in the outlet of both faucets, but it was not found in the surrounding environment. However, Pseudomonas aeruginosa was detected in the water sample of the water dispenser of Zhongshan Hospital of Traditional Chinese Medicine, Guangdong Province. The investigation found that the water dispenser was placed next to the sink, so it was suspected that biofilm was formed inside the faucet, and the colonization bacteria accompanied the droplets splashing through hand washing activities, which led to the contaminate of the facilities around the faucet^18^.

Pseudomonas aeruginosa was detected in two faucets sampled in this study. Looking back at all inpatients in two wards within one month, it was found that there was one patient infected by Pseudomonas aeruginosa in the ward where sink A was located, but it was not nosocomial infection. No patient infected by Pseudomonas aeruginosa was found in the ward where sink B was located. Although Pseudomonas aeruginosa colonized in two faucets has not caused an outbreak of nosocomial infection, it is very likely to lead to serious consequences if the water system is neglected to be cleaned and disinfected. Therefore, enough attention should be paid to the conditional pathogenic bacteria at the faucet outlet, and the cleaning and disinfection of water system should be included in the cleaning and disinfection of the sick room and disinfected regularly. In addition, the level of bacterial contaminate in and around the sink is related to the frequency of hand washing, and the bacterial content in the sink will increase with the increase of hand washing times. The contaminate of hand-washing facilities directly affects the hygiene quality, which is closely related to the occurrence of hospital infection^19^. Pseudomonas aeruginosa and Staphylococcus epidermidis are common opportunistic pathogens of hospital infection. We should attach great importance to the establishment of cleaning and disinfection system for hand hygiene facilities. We should thoroughly clean and disinfect the countertops, faucets, faucets handles, hand sanitizers, containers and other pollutants that can be seen by the naked eye at any time. When there are infectious diseases or blood and body fluids contaminate, we need to disinfect them after cleaning^20^. Hospital infection management department and all departments should regularly monitor and inspect the cleaning and disinfection effect of hand hygiene facilities in ward, find and solve problems in time, and prevent the outbreak and epidemic of hospital infection caused by hand hygiene problems; Higher administrative departments should also attach great importance to the contaminate of hand hygiene facilities and establish relevant monitoring norms and evaluation standards to guide and standardize clinical work. In short, safe hand hygiene facilities are the guarantee of hand hygiene effect.

There are many potential transmission sources in the hospital environment, and the actual source of pathogenic bacteria is usually difficult to determine, and there are environmental strains unrelated to the outbreak. In order to clarify the outbreak source and control the outbreak in time and accurately, genetic typing and homology analysis of the outbreak strains are important investigation methods. Retrospective investigation found that there was no outbreak of hospital infection in two wards within one month, and at the same time, due to funding constraints, the gene sequencing analysis of bacteria detected on the surface of objects and bacteria detected on the surface of faucets was not carried out in this study, which needs further analysis in future research.

## Data Availability

Routine surveillance data cannot be shared publicly because the provision of the data is dependent on the intended use. All the data supporting the findings of the work are contained within the manuscript (see Table 1 and Table 2).
